# Policing the pandemic: Frontline officers’ perspectives on organisational justice

**DOI:** 10.1177/14613557221132492

**Published:** 2022-11-01

**Authors:** Denise Martin, Neil Leslie, William Graham

**Affiliations:** Division of Sociology, School of Business Law and Social Sciences, Abertay University, UK; Division of Sociology, School of Business Law and Social Sciences, Abertay University, UK; Division of Sociology, School of Business Law and Social Sciences, Abertay University, UK

**Keywords:** Pandemic, organisational justice, organisational climate, front line, learning from crisis

## Abstract

Much of the literature on the policing of the coronavirus pandemic reflects research that has been extra-organisationally focused, examining the prospective impact of the police approach to applying the public health legislation on relationships with the public and the potential impact on police legitimacy. Less research has been intra-organisationally focused; investigating the potential affect on police officers of policing during an extraordinary public health crisis, which has required them to navigate an ambiguous and constantly fluctuating legislative and policy landscape that has driven significant changes to internal working practices and operational procedures within their organisational environment. Using original empirical data from a small multi-method study within one Division of a United Kingdom police force, we examined the issue from an organisational justice perspective; exploring perceptions of intra-organisational fairness, and how these may have directly impacted upon the responses of frontline officers during the pandemic. We argue that in this period, both organisational processes and their resultant outcomes did not meet normative expectations, as they discriminated against officers with public-facing (frontline) roles. This directly impacted upon the officers’ experience of the ‘organisational climate’. The resulting sense of organisational injustice felt by frontline officers reduced their morale, impacted upon relationships with senior officers, and nurtured feelings that they were not being treated with respect, dignity and trust. We conclude by discussing the potential implications of the study for police organisations and their leaders, outlining opportunities for organisational learning and consider the need for the development of policy that complements notions of organisational justice.

## Introduction

In early March 2020, the World Health Organization declared the novel coronavirus (COVID-19) outbreak as a global pandemic. The COVID-19 crisis went on to have a profound effect on policing in the United Kingdom (UK) and beyond (Maskály et al., 2021). However, even before the current public health crisis, the contemporary policing environment had been identified as particularly challenging; one of seemingly constant organisational change and reform, coupled with increasing public demand and media scrutiny (Newiss et al., 2021). Historically identified as an occupation that is extremely stressful and exacting under normal circumstances, Collins and Gibbs (2003) highlight that alongside everyday operational stressors, internal organisational issues often compound police officers’ stress; directly impacting upon their sense of wellbeing, mental health and resilience. Previous research has identified strain caused by perceptions of injustice within the policing organisation itself may affect the ability of officers to operate effectively. Wolfe and Piquero (2011) highlight that when officers sense unfairness within organisational processes and procedures, and in the decisions and actions of managers, they may be more likely to reject core organisational values, such as the principles of procedural justice.

Police officers, as first responders during the COVID-19 pandemic, were charged with performing a unique and uncertain public-facing role (Kyprianides et al., 2021). Public health legislation criminalised previously lawful, mundane everyday social activities. Sheldon (2021) highlights that officers were tasked with interpreting and enforcing the severest restrictions placed on democratic rights and liberties in modern times. Generally law-abiding citizens became subjects of police scrutiny and investigation, and although much attention has focused externally on the potential damage the police approach could cause to public trust and confidence (Jones, 2020), less has concentrated internally upon how frontline officers were expected to accept and adapt to the fundamental changes to their everyday role; changes exacerbated by an ambiguous, rapidly changing legislative and policy landscape, a reconfiguration of workplace and working arrangements and a highly increased likelihood of infection.

Within the UK, against a backdrop of media and public concerns about the initial police response to enforcing austere and untested legislation, the College of Policing and the National Police Chiefs Council (NPCC) developed joint guidance promoting a four Es (engage, explain, encourage, enforce) policing approach to public engagement. Reinforcing the principles of the UK's consensual policing philosophy, the approach emphasised the importance of fairness and justice in officer behaviours when interacting with citizens (College of Policing, 2021). Although the public-facing elements of the pandemic policing approach provide overt operational representations of procedural ethics, fairness and justice in action, Kyprianides et al. (2021) conclude that effective, clear and fair intra-organisational communication, leadership and management were not only critical to the successful delivery of the four Es approach specifically, but also to whether officers invested in democratic forms of policing generally. This refers to the process of organisational justice.

Complementary to procedural justice, the concept of organisational justice is concerned with intra-organisational notions of fairness, trust and equity: the extent to which employees perceive organisational policies, procedures, decisions and outcomes to be fair and just (Roberts and Herrington, 2013). Within policing, Schafer (2013) argues that trust and transparency between decision-makers and junior officers influence not only levels of organisational justice internally, but also those of procedural justice externally. Schafer contends that within organisations generally, trust permits employees to have the conviction that those in power will strive for ethical and considered conclusions to issues; that they will foster a working relationship, within which employees view them as honest, impartial and honourable; and that they will make decisions that are consistent and based upon sound judgement. This position is supported by a meta-analysis conducted by Wolfe and Lawson (2020), who conclude that organisational justice is a key predictor of critical outcomes among criminal justice system employees. Workman-Stark (2021) argues that creating a just organisational climate is essential for maintaining individual employee satisfaction and ensuring organisational effectiveness, particularly during periods of uncertainty. The extraordinary policing situation arising as a consequence of the pandemic presented a uniquely unpredictable and challenging environment for police practitioners; one within which police organisations may have had trouble in observing or maintaining principles that create the sense of a ‘just climate’ organisationally.

Drawing on empirical data from a study with one police Division of a UK-based police service, this article assesses the challenges of operating within the unique and continually fluctuating policing landscape created because of the COVID-19 pandemic. We argue demarcations between public health legislation and advice were often vague, inconsistently communicated and frequently conflated; and combined with fundamental changes to working arrangements and practices; this resulted in a perceived deficit of organisational justice for frontline officers during the pandemic. Moreover, it is argued that the sense of organisational injustice may have directly impacted officers’ attitudes and opinions of the organisational handling of the crisis. We conclude by arguing that as the UK emerges out of the pandemic these changes that occurred may have longer terms consequences for the police as a service.

## A climate of organisational justice

Shale (2020: 2) argues that ‘when we learn that we cannot rely on each other, or authority, to uphold fundamental normative expectations we suffer a loss of trust and confidence in people, principles, rules, processes, and institutions’. This quote, connected to the concept of moral injury, illustrates sentiments similar to those underpinning notions of organisational justice or rather injustice. In relation to policing, although procedural justice is seen as critical to maintaining and enhancing police legitimacy (Tyler, 2003), it is argued that the ability of police officers effectively to respect and observe procedurally just actions is determined by their experience of internal mechanisms and processes (Roberts and Herrington, 2013; Schafer, 2013; Van Craen and Skogan, 2017).

Conceptually, as Roberts and Herrington (2013) argue, organisational justice and procedural justice share the common principle of being concerned with the perceptions of fairness and outcomes that emerge from interactions between individuals. The concepts differ insofar as organisational justice reflects inwardly on matters that are intra-organisational and is concerned with how fair employees feel their treatment by authority figures and peers within an organisation is. Conversely, procedural justice focuses outwardly on extra-organisational relationships, and relates to the perceived fairness of strategies, policies, procedures and behaviours as they apply to stakeholders out with an organisation. In policing terms, the most obvious external stakeholders are ‘the policed’. Schafer (2013) asserts that there are distinct parallels between extra-organisational and intra-organisational notions of justice, as they are germane to ideas of consent. Just as officers rely upon consensual citizens for their authority to police, police leaders depend upon consensual officers for their authority to lead. Schafer adds that procedurally just public-facing policing is not possible without an organisationally just internal environment.

To a certain extent police officers manage their external environment more effectively because of experience, training and autonomy (Schafer, 2013). However, within the internal organisational environment, because police leaders are responsible for directing organisational effectiveness from the top down, they principally control the formalisation of policies and procedures (Stevens, 2017). Consequently, as Russell (2014) highlights, this may lead to frontline officers feeling unfairly excluded from decision-making processes, and unable to properly challenge directives that impact upon everyday frontline policing. Frontline officer perceptions that police leaders are inclusive in decision-making processes are likely to promote notions of organisational identity and empowerment; commitment to organisational objectives; and positive outcomes in interactions between officers and citizens (Williams and Cockcroft, 2019). Therefore, it could be argued that organisational justice transcends perceptions of fairness, because it is also linked to both group membership, and social identity. This is highlighted by Aston et al. (2021), who suggest that a more dynamic model of organisational justice must reflect interactive cultural processes embedded within policing. Therefore, organisational culture may be seen to either promote or frustrate the realisation of organisational justice within police agencies (Van Craen and Skogan, 2017).

Although conceptualisations of organisational justice have developed and changed over time, four closely related dimensions are generally understood to be the key constituents of organisational justice (Colquitt, 2001). First, distributive justice, which relates to equity in the distribution of outcomes to staff, such as fairness in salary, incentive and discipline distribution (Fridell et al., 2021). Cohen-Charash and Spector (2001) highlight that in original studies of organisational justice, perceived distributive injustice was found to have a negative impact on the cognitive and emotional state of employees, resulting in decreased workplace attendance and performance. Second, procedural justice, which refers to the organisational processes and procedures through which outcomes are realised, but not necessarily to the outcomes themselves, even though they may be individually unfavourable: the notion that the fairness of the procedure leading to the outcome is more important than the outcome itself (Ledimo, 2015). In instances of perceived procedural injustice, rather than the unfairness manifesting itself in individual dismay, the resulting negativity and sense of injustice is directed towards the organisation (Cohen-Charash and Spector, 2001). Baldwin (2006) argues that staff notions of procedural justice are likely to be augmented if decision-makers observe the ‘voice principle’: namely, that staff are presented with an opportunity to voice thoughts and concerns with leaders prior to the decision. Third, interactional justice, which relates to the interpersonal aspects within an organisation, and to the degree in which employees perceive those leaders treat them fairly, honestly and respectfully (Fouquereau et al., 2020). When employees sense that they are treated fairly in interactions with decision-makers, they are increasingly likely to feel positive about the organisation, and commit to organisational values, principles and rules (Quinton et al., 2015; Van Craen and Skogan, 2017). Fourth, informational justice, which relates to the transparency and clarity with which leaders communicate their decision-making rationale and the distribution of the decision's outcomes to people within the organisation (Colquitt et al., 2001; Roberts and Herrington, 2013). In work with specific regard to policing, Gau and Gaines (2012) found that explaining top-down decisions and providing junior officers with the opportunity for feedback were more likely to lead to successful outcomes. Van Craen and Skogan (2017) highlight the radical police reform agenda in the United States, aimed at increasing public trust and confidence, which emphasises the centrality to reform of embedding procedurally just organisational processes while acknowledging that to do so requires changes to organisational culture and structures. Before considering this in more detail through examination of the data, we outline our methodological approach.

## Methodology

This research was initially carried out with one Division of a UK police service. The Division covers a large geographical area and includes both rural and densely populated areas, meaning that during the pandemic officers were likely, depending on their role, to be operating in what were previously busy city areas, as well as quieter community locations.^1^

A multi-method qualitative research model was designed using the combination of a survey questionnaire, focus groups and semi-structured interviews to collect and triangulate the data. There were two phases to the research. A survey questionnaire, posing several open and closed questions, which was prepared using the Google Forms survey platform and circulated electronically in February 2021 via the internal email system of the police force. Questions were devised in line with the original research aims and objectives of the study, which were to identify the learning that the police service could take from their response to the COVID-19 crisis, including examining the processes and systems implemented by the police to reflect and manage organisational responses during the pandemic, and to understand practitioner and leadership teams’ experiences of policing during this period. The questionnaire was aimed at frontline staff and first-line supervisors. We had a single point of contract (SPOC) within the force who acted as a gatekeeper for the research. Our position as previous police researchers known to key contacts within the service probably assisted in supporting access. The SPOC supported the distribution of the questionnaire across the Division; and responses were returned directly to the research team, via a secure portal only accessible by the team. Ethical approval was obtained through the internal university process and the universitiy's General Data Protection Regulation (GDPR) policies and procedures were followed. Participants were provided with participant information sheets and informed consent was obtained from all participants; for online methods this was done via email. The questionnaire is believed to have reached approximately 200 staff members. Seventy-seven responses were received, which equates to a response rate of roughly 39%.

In the second phase of the project in March 2021, which consited of semi-structurd interviews and focus groups, our SPOC requested volunteers to participate in the research, therefore, officers were self-selecting to some extent. This can be viewed positivly as officers were not pressurised by the organisation to participate and might mean we were given a more realistic account of their experience. However, it might mean that officers who had a negative view of the organisation might have felt more compelled to participate. Getting an accurate picture of the organisation is always a dilemma for police researchers (Burger, Tong and Martin 2016). Our choice of a mixed-method design hopefully helped to prevent this to an extent.

Three focus group sessions were held with frontline officers and their supervisors: one comprising solely of constables, one comprising solely of sergeants, and one comprising both constables and sergeants of the Special Constabulary. Because of national COVID restrictions at the time all interviews and focus groups were conducted using the Microsoft Teams meeting platform and audio visually recorded. The number of participants in each focus group was kept to a small number with each containing approximately three to five participants. Lobe et al. (2021) suggest that smaller focus groups work more effectively when using online interviewing platforms. Semi-structured interviews were also undertaken with eight staff members holding representative, managerial and strategic positions within the force. The interviewees comprised one superintendent, two chief inspectors, three inspectors, a police federation representative and a senior member of police support staff. Transcriptions were later prepared by an approved independent transcriber.

## Thematic analysis

Thematic analysis was used to analyse the data; this was seen as the best way to identify, analyse and interpret the findings (Braun and Clark, 2006). Initial themes were developed applying an inductive approach and reading of the qualitative comments in the questionnaire and from the interview and focus group transcripts. These included organisational learning, officer safety and welfare, organisational practice, leadership, communication, logistics, valuing staff and leadership. Once key themes were established, scripts were re-read and subsequent data coded and refined according to these themes.

## Officer safety

Shale (2020) argues that employees may incur ‘moral injury’ when employers fail to satisfy the normative expectations of employees. The resulting employee resentment can be organisationally positive, because it may serve to remind employers of how employees distinguish moral boundaries within the organisation. However, the resentment may be organisationally harmful when it festers and becomes a source of ongoing bitterness and disillusionment. In the context of policing, at the beginning of the public health crisis, an example of failing to meet normative expectations was the perceived organisational inadequacies in the provision of personal protective equipment (PPE). De Carmargo (2021) in her study on the provision of PPE found that the sudden lack of availability of PPE exacerbated officers’ feelings of apprehension. Frontline officers’ anxiety regarding the organisation's inability to meet normative expectations in respect of PPE supply was a recurrent ‘officer safety’ concern identified within this study's data also. Although concerns were not universal, with some frontline officers reporting a speedy and efficient process, the following quotes from frontline and Special Constabulary officers should be considered in the context that they were made almost 12 months after the legislation restricting social activities was enacted. Supporting Shale's (2020) ‘festering resentment’ argument, the data suggest that although initial PPE supply issues were resolved by the force concerned, the sense of resentment lingered and continued to be evident long after the initial problem had been addressed by leaders:

We didn’t have any, basically. I mean, face coverings … we did get some through after three weeks, but we initially got, I think, three (paper masks) per officer, and that was it. Three per officer and they’re only meant to last an hour and a half. (Frontline officer)

To be honest, the whole PPE scenario and training was just abysmal, it took far too long to sort out from people who are in really senior levels. (Senior officer)

[A] lot of Special Constables reported not receiving any [PPE] … the vast majority of station commanders only requested PPE for the regular officers and forgot to include Special Constables … massive, massive feelings of undervalued, devalued, do you know. You’re just a Special. (Special officer)

Resentment was echoed in other frontline officer comments about PPE and officer safety more broadly; not in relation to the availability and issue of PPE to officers, but in respect of how officers perceived that the force's ‘public image’ was being prioritised over officer safety. In the early weeks of mandated social restrictions, there remained uncertainty surrounding levels of infection protection provided by face coverings, at a time when the wearing of them in public had not yet been mandated. Of course, the choice of many citizens was to adopt a mask-wearing regime as part of what they considered sensible precautionary measures. Against a backdrop whereby many staff, including most senior officers were already primarily working from home, frontline staff were initially instructed not to wear masks when on public-facing duties. The sense of frontline officer resentment engendered by this instruction is reflected in a range of data from the survey including the following quotes.

[I]n terms of the officer safety, initially we were told not to wear masks because of the public perception and how that might look to the public. (Frontline officer)

Officers safety came second to public perception. (Frontline officer)

In fact, a (location) cop actually got pulled up because he wore a face mask when we were told not to … now we’re actually getting pulled up for not wearing a face mask …. Because we didn’t want to scare … didn’t want to scare the public. Well, it was all over the news that there was a pandemic happening, so when you think if the police wear face masks, then it's a measure to say, ‘Look, the government's taking it serious’, and yet we were told not to. (Frontline officer)

It appears frontline officers felt a sense of organisational injustice across a range of issues related to PPE. Initially, they perceived procedural injustice because the organisational processes for providing them with the protective accoutrements required to ensure officer safety were inadequate and inefficient. Compounding this feeling of injustice, frontline officers sensed that organisational leaders had based decisions, prohibiting the wearing of face masks during public-facing duties, on the premise that citizens may not be receptive to this image. This resulted in the frontline perception that public sensibilities had greater importance than officer safety; frontline officers believed the process determining this outcome to be unjust.

## Leadership

Leaders have a critical role to play in nurturing and preserving a sense of organisational justice within junior staff because they can be viewed as an immediate source of information upon which employees base their views of organisational objectives and policies (Workman-Stark, 2017). Research has demonstrated that police officers who believe decision-makers apply organisational justice principles have higher levels of job satisfaction and possess greater trust in their agency (Wolfe and Piquero, 2011). More specifically, as identified by Dirks and Ferrin (2002), employees’ trust in leaders is influenced by perceived levels of fairness or justice in organisational practices and decision-making, because these are likely to be viewed by employees as indicative of their relationship with the leader, and the leader's character. In his development of the work-relations framework to understand police officers’ trust in citizens and supervisors, Van Craen (2016) draws on several theoretical frameworks and empirical insights to understand the origins and consequences of police officers’ trust and the roots of officers’ trustworthy behaviour. In relation to the intra-organisational features required to create trust, Van Craen identifies four critical elements, including internal procedural justice. He emphasises that, like the approach citizens expect in their interactions with officers, officers themselves anticipate fair and respectful treatment in their relationships with leaders.

In our study, the leadership arrangements introduced organisationally in response to the public health crisis did not appear to be welcomed by frontline staff. Frontline officer perceptions of an unhealthy deficit in leadership visibility were evident from our data, with a high number of participants rejecting the notion that effective leadership was possible while senior officers were working predominantly from home. Many frontline staff seemed frustrated, disappointed and resentful at what they perceived as an absence of visible leadership support and guidance from managers (inspectors and above, but particularly chief inspectors and above) during an extraordinarily challenging period for frontline policing:

How can officers hold confidence when their higher ups don’t stand with them? Don’t visit and stand in solidarity with the fellow officers. They work from home in a safe environment. Unfortunately, it's the boots on the ground that keep the ball rolling and the force working. (Frontline officer)

When hearing that members of the SMT (senior management team) are working from home, whilst officer levels were down due to self-isolation was extremely disheartening. (Frontline officer)

Having senior management in the office and contactable is a positive thing for us … we’re very much just left out here on our own quite a lot of the time. So, if you need a senior management decision made for something like a serious incident, you’ve really got to struggle to try and get in contact with someone. (Frontline officer)

The data indicate that frontline officers perceived that the decision-making process leading to senior officers being permitted to work from home was procedurally unfair and resulted in a distribution of outcomes that was also unjust. Frontline officers felt that there was a distinct disbenefit to them arising from the decision to allow senior officers (and indeed other non-frontline officers and police staff) to work from home, while conversely those able to do so benefited from greater flexibility in supporting families, and a vastly reduced risk of infection. It is unclear from the data whether there was a chief officer level directive that senior officers must work from home wherever operationally feasible; however, the data do indicate that such decisions were left to individuals to make:

There's not really been a guidance on that, to be honest … I’ve just taken it upon myself … I’ve just justified that I’ve not gone because I don’t see the risk of me going round three or four offices … I just think it's too great a risk, so I’ve just justified it myself, that I’m … minimum contact. (Senior officer)

[I]t's (working from home) back down to your own judgement and what you consider appropriate. (Senior officer)

Although often conflated with public health guidance and political messaging, the legislative position during periods of the pandemic's strictest restrictions was clear: in order to lawfully leave home to travel to work, it had to be ‘not reasonably possible’ to work from home (Scottish Parliament, 2020; UK Parliament, 2020). The grounds that constitute necessity to travel to work are arguable, and whether the legislators intended the law to extend to warranted police officers is also a moot point. Nonetheless, in this study, frontline officers perceived distributive injustice as a consequence of their leaders enjoying outcome privileges that they could not. The sense of injustice was compounded by the perceived absence of transparency and dialogue (procedural injustice) with frontline staff about the potential impact of reducing levels of leadership visibility from the expected norms. This finding is somewhat validated by other data within the study, because some senior officers appeared embarrassed by the disparity in outcomes:

… and we can say that we’re open-door policy, all these things, nobody, well nobody is going to call out a senior officer and say, ‘Well, I actually think it's a bit ridiculous that you are all sitting in the house, and we’re out working’. (Senior officer)

The study data also revealed a notion within senior officers that paid overtime would somehow appease frontline officers’ pandemic policing anxieties:

The cops have never been so well paid … they’re making more money than the superintendent, so that's all I’ll need to tell you about the overtime they’re getting. (Senior officer)

In line with the principles of interactional justice, previous studies (for example, see Bell et al., 2015) highlight the need for careful management of frontline officers’ working hours, as increased time at work may result in fatigue, stress, insomnia, cognitive impairment and a reduction in commitment to organisational goals and imperatives.

In this study, the leadership perspective and style adopted by managers during the pandemic may have undermined frontline officers’ trust and weakened their faith in the integrity of their leaders. Van Craen (2016) argues that the behaviour of managers functions as an important signal to junior officers about the moral standard of the society within which they work. If trust in leaders is compromised, frontline officers are less likely to conform with organisational rules, reinforcing the negative behavioural aspects of police occupational culture identified by several police scholars (for example, see Cockcroft, 2012; Loftus, 2010). Drawing on management theory's ‘exchange relationship framework’ (Whitener et al., 1998), which conceptualises trustworthy management behaviours to be grounded on five key components (integrity, consistency, communication, concern and involvement), Van Craen (2016) posits that fostering positive organisational bonds requires police leaders to consider their relationship with junior officers to be one that is reciprocally beneficial. Pandemic policing's socially distanced forms of leadership may have decreased the likelihood of sustaining positive leader/officer relationships, and in this study appeared to result in a significant disconnect between frontline officers and their leaders. As identified in previous research on occupational police culture (Reuss-Ianni and Ianni, 1983), the consequence of such a disconnect will likely be the development of a ‘them’ and ‘us’ mentality between frontline and management officers.

## Internal communication

Within the study's policing division, technological forms of internal communication were increasingly heavily relied upon during the pandemic, and progressively became embedded as part of ‘business as usual’. Although data indicate that some found the expansion of e-communication methods useful, many frontline officers reported a sense of being organisationally detached and unsupported as they strove to unpack and comprehend untested legislation; and to distinguish their actual powers from enforcement strategies, public health guidance and political rhetoric. Frequently during the pandemic, officers were confronted with new information from within the organisation, which they report feeling somewhat overwhelmed by. Our data indicate a frontline perception that exclusively electronic means of communicating critical information provided them with little or no opportunity to clarify decisions or raise concerns:

Sending out vague emails isn’t helpful. More face to face, detailed, training and guidance. I don't think there's ever been any proper briefing in relation to the sort of legislation that was brought in by the government. (Frontline officer)

For those at more senior levels to hear they need to speak to us more and engage with us more. (Frontline officer)

So, I think there was a bit of confusion, a lot of confusion with the cops, I think there was a bit of information overload at times. (Senior officer)

(internal e-communication) sites were just getting overloaded, and overloaded by, you know, everyday there was a new message would come out, a new instruction would come out. (Senior officer)

Particularly in the earlier part of the pandemic, as the legislative and policy landscape regularly fluctuated, understandable difficulties and delays were experienced in organisationally condensing and communicating messages regarding operational policy to frontline staff. Consequently, frontline interpretations were at times adopted from media and political sources, with public guidelines being conflated with legislative powers. Within this ambiguous operating environment frontline staff had a normative expectation that electronic means of communicating information should be supported by messages delivered via physical leadership engagement. Frontline officers perceived that using e-communication methods alone to deliver such critical information was disrespectful, and inadequate for their operational and emotional needs, causing additional anxiety and fuelling a sense of interactional injustice:

Lack of information from senior officers, same officers being invisible and not once attending briefings to provide information or reassurance. (Frontline officer)

I think sometimes, some of them (senior officers) forget just that we are on the ground and sometimes we just need that wee bit of extra, extra support and wee bit of encouragement from them. (Frontline officer)

… [T]here's no communication, like, from senior management in terms of, you know, something like this, you know, to ask how's things going? (Frontline officer)

The research data suggest a frontline belief that greater use of physical forms of leadership communication, complementing electronic information delivery, may have dissipated uncertainty and supported greater consistency and confidence in the application of equivocal legislative powers, policies and guidelines. The approach adopted by leaders within the police Division studied may have been deficient in terms of informational justice. Leadership may have believed that broad internal electronic circulation of the four Es policy approach provided sufficiently clear frontline guidance; however, the frontline officers in this study appear to reject that notion, feeling that organisational decisions were often not clearly relayed, which may have resulted in heightened senses of injustice and disengagement.

## Working arrangements

New compulsory social restrictions compelled employers worldwide to make significant changes to employees’ working arrangements and to reimagine what may constitute an effective employee workplace (de Lucas Ancillo et al., 2021). The changes applied similarly to police agencies with a reconfiguration of working practices taking place across UK constabularies (Her Majesty’s Inspectorate of Constabulary and Fire and Rescue Services, 2021), including a move to home working for many officers and police staff. The changes required an accelerated shift to remote technological workplace solutions, including significantly expanding the use of virtual meeting platforms, which permitted briefings, meetings, training delivery and supervision to take place almost exclusively online. In this study, it appears that organisational decisions on the introduction of new working practices were not balanced against the potential impact of the changes, particularly on frontline officers, whose role was arguably the one most affected. This finding supports that of other occupational research conducted by Galanti et al. (2021), who argue that the pandemic effectively compelled organisational leaders to hastily introduce new working arrangements, such as home working, in an unplanned and chaotic manner, and without ensuring employees were equipped with the necessary skills to adapt to the change.

In this study, frontline officers reported feeling that the new working arrangements, particularly home working, disfavoured them to the benefit of non-frontline personnel, and did not therefore represent a fair distribution of outcomes. This was compounded by a genuine sense of injustice, fuelled by the perception that decision-makers, who could generally work from home in relative safety, did not fully appreciate the feelings of anxiety felt by frontline officers. This is illustrated in commentary data from frontline officers:

Since the pandemic, inspectors have been working from home a lot, this is annoying as everyone else has to come in and put themselves at risk. (Frontline officer)

Laptops sent by (HQ) for home working were retained by senior officers rather than being issued as required. (Frontline officer)

Yeah, so officers, for example, on restricted duties, that was a big one. Do we need to have them in the office? And initially, supervision locally said, ‘Yes, we want them in the office so we can supervise that they’re doing work’. Now, all they’re doing is some office-based enquiries. If those officers wanted to work from home, could they work from home? … [T]he division ordered 50 laptops … then proceeded to give them to all the managers, from inspector above … so that they didn’t have to work from the office. Which again, I can understand why maybe they wouldn’t want to work from the office, but when you’ve got officers who don’t need to be in the office, who could work from home, who are not contributing as much because they’re on restricted duties, why do they still need to come in the office and put themselves at risk? (Frontline officer)

The last quote demonstrates a clear sense of confusion arising from a lack of organisational transparency in respect of leadership decisions on the identification, suitability and prioritisation of staff for home working. This indicates a deficit of both distributive and informational justice: first, it appeared to frontline officers that home working, although available to some, was a privilege that they could not realise, and therefore an outcome not fairly distributed across the organisation; and second, the rationale provided by managers for such decisions was opaque and relayed in a manner that did not engender a spirit of trust.

The sense of organisational injustice in respect of the working arrangements during the pandemic extended to officers of the Special Constabulary (Specials), who perceived an absence of organisational acknowledgement that they perform an unpaid volunteer role, and are dependent upon other salaried forms of employment outside policing for their livelihood:

[M]y job (paid employment) know that when I go out policing, I’m putting myself at risk and they’re not very happy about that …. There's been no welfare queries whatsoever, from either Special or regular management … I feel like I am less valued than the regulars, very much so. (Special officer)

Despite arguably being among the most suitable for flexible working arrangements, the data from this study indicate that Specials felt denied consideration for roles other than those providing a visible uniform presence when required, and they were explicitly refused the opportunity to work from home:

No attempt at all to get Special Constables to complete their duties from home and they’re the ideal candidates … a lot of them could be taking statements … raising crimes at home, a lot of them could be progressing things at home. Even doing the mandatory learning. (Special officer)

(Division) had sent me up one of their own brand-new tablets. Within a week of having that, I got a phone call … from my regular senior management team, stating that I need to hand back the computer immediately, I’m not allowed to work from home. (Special officer)

(An identified officer above inspector rank) basically said, ‘Well, we’re losing you to working from home, you’re basically no benefit … You’re basically no benefit sitting at home. (Special officer)

The Specials in this study felt a deep sense of distributive injustice that some salaried frontline officers could be afforded the opportunity to work from home, whereas they as unpaid volunteers were denied that possibility. There was also a strong sense from Specials that the pandemic had marked the re-emergence of cultural antagonism towards them from officers of all ranks, and Specials perceived levels of marginalisation, disrespect and resentment from frontline officers that were not evident pre-pandemic:

But I think what COVID has done … it's allowed a rebirth of that anti-Special rhetoric. (Special officer)

I wouldn’t join (name of force) now. In fact, I’ve not enjoyed my time with (name of force) as a Special during this period. I’m just waiting for it (pandemic) to stop to see whether it gets any better. (Special officer)

[R]egular officers were telling Special Constables not to attend for duty, do not to come into the police office, we don’t want more people putting us at risk. And officers were getting told by their chain of command that they had to do their … minimum commitment. So, they were getting contrasting issues. (Special officer)

The Specials’ perceptions of both interactional and procedural injustice highlighted above were somewhat validated by other data within the study from frontline officers:

[S]o they’ll (Specials) show up and a sergeant, or an inspector, might not have any idea of what their capabilities are. So, you really have to be careful who they’re teamed up with, you know, what they can deal with. (Frontline officer)

And they’re now bringing in Specials which, yeah, Specials are great to have as an extra support but the job seems to have the Specials taking the placement of another officer. (Frontline officer)

Previous Special Constabulary research highlights the cultural antipathy and scepticism that frontline officers have displayed towards Specials historically. Bullock and Leaney (2016) stress that the effectiveness of Specials is largely dependent upon them being treated with respect and dignity, organisationally valued and integrated with their frontline colleagues. These are also considerable factors in the recruitment, morale and retention of Specials.

Overall, there was a distinct disbenefit to frontline officers and Specials from decisions allowing senior officers and other staff to work from home, because officers performing frontline duties could not avoid physically proximate interactions with citizens and colleagues, which exposed both them and their families to far greater risk of infection. Conversely, those working from home benefited from the ability to provide greater physical and emotional support to their families and enjoyed a vastly reduced risk of infection. Therefore, when senior officers were making decisions on whether or not they themselves could/should work from home, it is difficult to imagine that they were not faced with something of a moral dilemma: with a strong individual incentive to place personal ethics before professional.

## Discussion

Examining frontline officers’ experiences during the second UK ‘lockdown’ in the early part of 2021, this study's data indicate that the organisational climate was unable to provide officers with the levels of reassurance and support that may have been required in this space. The study acknowledges that the public health crisis has presented an unprecedented and extraordinary working environment for the police service and all other public sector bodies, which has significantly impacted upon their ability to operate effectively. Nevertheless, the study's findings do indicate that certain organisational decision-making processes and their outcomes, such as those pertaining to officer safety, leadership, internal communications and working practices, did not satisfy the normative expectations of officers required to perform frontline duties. It appears that these issues impacted negatively on the organisational climate within the police division concerned, increasing perceptions of organisational injustice.

Within the scope of the study, it was difficult to assess whether frontline perceptions of organisational injustice pre-date the pandemic, or whether pre-existing resentment has been exacerbated by the challenging public health-focused policing landscape. Nevertheless, in circumstances in which a negative organisational climate prevails, it is likely to have a direct impact on how officers operationalise their duties. Quinton et al. (2015) suggest that officers who have positive experiences of organisational fairness are less likely to be receptive to the cynicism and authoritarianism inherent in police subcultures; and more likely to embrace organisational values and goals, adopt ethical policing practices and exhibit procedurally just behaviours when interacting with citizens. Applied to the achievement of organisational objectives, this indicates the importance of creating a positive organisational climate, within which employees feel respected, valued and justly treated.

In this study, when decision-makers disregarded organisational justice principles, junior officers felt excluded, and the research data indicate a strong sense of poor morale and disillusionment among officers (both frontline and Specials) with public-facing responsibilities. These feelings may have been aggravated by other pandemic policing challenges, not least of which was the significantly increased exposure to the risk of infection from routine interactions with colleagues and the public. Other research (for example, see Frenkel et al., 2021; Stogner et al., 2020) highlights that frontline officers experienced increased levels of stress as a consequence of policing during the pandemic.

On a general note, police forces may wish to consider whether there is intra-organisational learning that can be extracted from their performance during the pandemic. The findings of this small study indicate that to identify learning points, forces may need to evaluate the strategies they adopted during the pandemic and the processes involved in arriving at decisions effecting policy; and to reflect upon the outcomes for public-facing officers that were realised as a consequence because these may have impacted upon the organisational climate. Research examining the organisational effect of the coronavirus pandemic on police agencies globally (Maskály et al., 2021) suggests that police executives are, arguably surprisingly, relatively unconcerned regarding the longer-term strategic impact within agencies, with strategic leaders proposing that few negative intra-organisational outcomes have been caused by policing the pandemic. Based on the findings from our study, such a proposition appears overly optimistic, particularly considering the scale of the changes organisationally, and the often frenetic, unpredictable and chaotic policing environment within which the changes were introduced.

Findings from other research (for example, see Brown and Fleming, 2021) suggests frontline staff were critical of pandemic-related organisational processes and outcomes during the crisis. The perceived injustices of public-facing officers from the data in our small study support these findings. Given that such feelings of disapproval are likely to contribute to a negative organisational climate, police leaders may wish to take steps to establish the current organisational climate within their own forces, and in cases in which frontline trust in organisational justice has deteriorated as a consequence of pandemic policing requirements, explore how this can be restored as policing re-emerges from the COVID crisis. Furthermore, there may be a need for police organisations to embrace critique from frontline officers and acknowledge the learning that may emanate from their experiences of policing the pandemic. Hartmann and Hartmann (2020) argue that frontline innovations and ideas for improvement frequently emerge during crises, and although strategic level learning is important, the learning that can be extracted from frontline concerns and experiences is invaluable, and is often lost, ignored or left unexploited. Left unaddressed, it is improbable that perceptions of organisational injustice within frontline officers would dissipate. The resulting disillusionment and alienation would be likely to prevent the identification of innovative frontline suggestions, and therefore frustrate organisational learning as police services emerge from the public health crisis.

### Limitations

The findings of the study were drawn from a small sample of officers from one police Division in the UK, therefore this study cannot claim to be generalisable to all police forces, or to the experiences of officers policing the pandemic in other locations; however, its findings tend to support those of other recent research (Brown and Fleming, 2021; De Camargo, 2021; Fleming and Brown, 2021; Frenkel et al., 2021; Stogner et al., 2020), which is indicative that the experiences of officers within this study may be replicated across police organisations more broadly. This study allowed frontline practitioners to provide a narrative about their direct experiences, feelings and perceptions of working during a period of crisis (COVID-19) and introduced the organisational justice framework as a lens through which to examine and seek to understand how they coped with policing that crisis. The findings can contribute to ongoing debates about the imperative of internal justice within police organisations in order to promote the public-facing external forms of justice required to sustain police legitimacy.

## Conclusions

This small study considered the intra-organisational experiences of frontline police practitioners during the coronavirus pandemic by analysing empirical data from staff within one division of a UK police force. The study's data indicate that decision-makers may not have adequately observed the key principles of organisational justice in terms of the fairness of processes and procedures, distribution of outcomes, interactions and transmission of information. Consequently, officers with public-facing duties felt particularly disadvantaged and aggrieved. In the absence of the ability to effectively pre-plan, we argue that organisational responses during dynamic major crises must still be carefully considered before being implemented. In instances in which frontline officers perceive organisational decision-making processes and outcomes to be unjust, they are more likely to adopt the negative traits historically associated with occupational police culture, and less likely to absorb organisational aims or exhibit ethical behaviours within public interactions. This would have a potentially negative impact on citizens’ trust and confidence in the police and on their consent for the legitimate authority of officers. The issue of organisational justice is an ongoing one that police agencies may wish to monitor; and consider how it can be sustained and developed to ensure a healthy organisational climate as policing migrates to what is hopefully the post-pandemic period.

## Notes

1. Owing to the potential for revealing the identity of the division and force, we can provide no further details to protect the anonymity of our participants and the service who authorised access to complete the research.
